# Supplementary material for: Empirical methods for controlling false positives and estimating confidence in ChIP-Seq peaks
Source: BMC Bioinformatics. 2008 Dec 5;9:523. doi: 10.1186/1471-2105-9-523 (PMC2628906; doi:10.1186/1471-2105-9-523)
Supplement: Additional File 1 — A variety of html documents from the USeq web site detailing the available applications, their best usage, output file type descriptions, command line menus, etc. [file 1471-2105-9-523-S1.zip › USeqUserGuides/JavaDoc.zip!/JavaDoc/trans/main/SamChromosomeData.html]

SamChromosomeData


|  |  |  |
| --- | --- | --- |
| **Overview** \| **Package** \| **Class** \| **Use** \| **Tree** \| **Deprecated** \| **Index** \| **Help** | |  |
| **PREV CLASS**   **NEXT CLASS** | **FRAMES**    **NO FRAMES**     **All Classes** |  |
| SUMMARY: NESTED \| FIELD \| CONSTR \| METHOD | DETAIL: FIELD \| CONSTR \| METHOD |  |


---


## trans.main Class SamChromosomeData

```
java.lang.Object
  trans.main.SamChromosomeData
```

---

``` public class SamChromosomeData extends java.lang.Object ```

---

**Constructor Summary**

`SamChromosomeData(int numberOfWindows,
int numberOfPermutations)`


| **Method Summary** | |
| --- | --- |
| `void` | `calculateFudgeFactor(int windowIndex)` |
| `float[][]` | `getPermMeanDiff()` |
| `float[][]` | `getPermTStats()` |
| `float[][]` | `getPermVariance()` |
| `float[]` | `getRealMeanDiff()` |
| `float[]` | `getRealTStats()` |
| `float[]` | `getRealVariance()` |
| `void` | `setPermMeanDiff(float[][] permMeanDiff)` |
| `void` | `setPermSamScores(float[] samScores, int permutationIndex, int windowIndex)` |
| `void` | `setPermTStats(float[][] permTStats)` |
| `void` | `setPermVariance(float[][] permVariance)` |
| `void` | `setRealMeanDiff(float[] realMeanDiff)` |
| `void` | `setRealSamScores(float[] samScores, int windowIndex)` |
| `void` | `setRealTStats(float[] realTStats)` |
| `void` | `setRealVariance(float[] realVariance)` |

**Methods inherited from class java.lang.Object**

`equals, getClass, hashCode, notify, notifyAll, toString, wait, wait, wait`

**Constructor Detail**

### SamChromosomeData

```
public SamChromosomeData(int numberOfWindows,
                         int numberOfPermutations)
```


**Method Detail**

### setRealSamScores

```
public void setRealSamScores(float[] samScores,
                             int windowIndex)
```

---


### setPermSamScores

```
public void setPermSamScores(float[] samScores,
                             int permutationIndex,
                             int windowIndex)
```

---


### calculateFudgeFactor

```
public void calculateFudgeFactor(int windowIndex)
```

---


### getPermMeanDiff

```
public float[][] getPermMeanDiff()
```

---


### setPermMeanDiff

```
public void setPermMeanDiff(float[][] permMeanDiff)
```

---


### getPermTStats

```
public float[][] getPermTStats()
```

---


### setPermTStats

```
public void setPermTStats(float[][] permTStats)
```

---


### getPermVariance

```
public float[][] getPermVariance()
```

---


### setPermVariance

```
public void setPermVariance(float[][] permVariance)
```

---


### getRealMeanDiff

```
public float[] getRealMeanDiff()
```

---


### setRealMeanDiff

```
public void setRealMeanDiff(float[] realMeanDiff)
```

---


### getRealTStats

```
public float[] getRealTStats()
```

---


### setRealTStats

```
public void setRealTStats(float[] realTStats)
```

---


### getRealVariance

```
public float[] getRealVariance()
```

---


### setRealVariance

```
public void setRealVariance(float[] realVariance)
```


---


|  |  |  |
| --- | --- | --- |
| **Overview** \| **Package** \| **Class** \| **Use** \| **Tree** \| **Deprecated** \| **Index** \| **Help** | |  |
| **PREV CLASS**   **NEXT CLASS** | **FRAMES**    **NO FRAMES**     **All Classes** |  |
| SUMMARY: NESTED \| FIELD \| CONSTR \| METHOD | DETAIL: FIELD \| CONSTR \| METHOD |  |


---
